# Surgical Treatment of Concomitant Atrial Fibrillation: Focus onto Atrial Contractility

**DOI:** 10.1155/2015/274817

**Published:** 2015-07-01

**Authors:** Claudia Loardi, Francesco Alamanni, Claudia Galli, Moreno Naliato, Fabrizio Veglia, Marco Zanobini, Mauro Pepi

**Affiliations:** Department of Cardiac Surgery, Centro Cardiologico Monzino, IRCCS, University of Milan, Via Parea 4, 20138 Milan, Italy

## Abstract

*Background*. Maze procedure aims at restoring sinus rhythm (SR) and atrial contractility (AC). This study evaluated multiple aspects of AC recovery and their relationship with SR regain after ablation. *Methods*. 122 mitral and fibrillating patients underwent radiofrequency Maze. Rhythm check and echocardiographic control of biatrial contractility were performed at 3, 6, 12, and 24 months postoperatively. A multivariate Cox analysis of risk factors for absence of AC recuperation was applied. *Results*. At 2-years follow-up, SR was achieved in 79% of patients. SR-AC coexistence increased from 76% until 98%, while biatrial contraction detection augmented from 84 to 98% at late stage. Shorter preoperative arrhythmia duration was the only common predictor of SR-AC restoring, while pulmonary artery pressure (PAP) negatively influenced AC recuperation. Early AC restoration favored future freedom from arrhythmia recurrence. Minor LA dimensions correlated with improved future A/E value and vice versa. Right atrial (RA) contractility restoring favored better left ventricular (LV) performance and volumes. *Conclusions*. SR and left AC are two interrelated Maze objectives. Factors associated with arrhythmia “chronic state” (PAP and arrhythmia duration) are negative predictors of procedural success. Our results suggest an association between postoperative LA dimensions and “kick” restoring and an influence of RA contraction onto LV function.

## 1. Introduction

AF presents with different frequencies in patients affected by structural heart diseases requiring surgery, showing a peak incidence of up to 60–80% in mitral subjects [1]. It causes an increased risk of systemic embolism, cardiac failure development, and higher limitations due to dyspnea and fatigue on exercise. Cox-Maze technique for surgical AF treatment started being used in 1987 [2] and it has suffered numerous modifications since then. Nowadays, most centers have replaced the “cut and sew” technique by other methods using several power sources to achieve the same target in a much easier way [3]. One of the alternative energy sources currently used is radiofrequency (RF) ablation which has been demonstrated to be a simple, safe, and reproducible procedure with an acceptable success rate in terms of SR restoration [4].

The original Maze was designed with three specific goals in mind: (1) the permanent AF ablation, (2) the restoration of atrioventricular synchrony, and (3) the preservation of atrial transport function [2]. If, by one side, the efficacy of the procedure in reaching the first two goals is widely known [5, 6], the restoration of the SR does not always accompany the corresponding recuperation of atrial mechanical “kick” [7, 8]. Atrial contraction is effective when A waves are found in tricuspid and/or mitral transvalvular flow using Doppler echocardiography [9], allowing the patient to fully profit from hemodynamic and clinical advantages of an organized atrial contraction. If the atrial transport function fails to recover, benefits deriving from arrhythmia abolition might only be marginal, since, by one side, blood stasis in the atria persists, thus maintaining unchanged thromboembolic risk and, by the other side, heart hemodynamic performance is still impaired resulting from the loss of atrial contribution to cardiac output [10].

Despite its relevant role for judging Maze comprehensive success, biatrial contractility restoration and its clinical importance have not been deeply investigated. In more detail, a parallel deep analysis and comparison of possible determinants of both surgical ablation tied goals (AC and SR achievement) is still lacking. In the present study, we describe 2-year results of concomitant modified RF Maze in a homogeneous population of mitral patients, focusing onto common predictors of SR and AC restoring and their time-course appearance and possible relationship with cardiac chambers dimensions and function evolution.

## 2. Methods

### 2.1. Patients' Characteristics

We analyzed a total of 122 consecutive patients with mitral valve disease scheduled for cardiac surgery who underwent a modified RF Maze procedure between January 2005 and June 2012 at our center (Table 1). There were 59 males and 63 females, with a mean age of 62 ± 8.5 years (range 30–82 years) at the time of operation. 64.7% of patients had permanent or persistent AF with a mean preoperative duration of 28.3 months (range 1–220 months); in the others our indication to perform the concomitant RF was an at least double occurrence of paroxysmal AF refractory to medical treatment (arrhythmia definitions based on ACC/AHA Guidelines 2011) [11].

Mean preoperative LA dimensions were 56 ± 12 mm and 34 ± 7 cm^2^. Mean basal PAP was 33 ± 15 mmHg. Patients experiencing mitral repair with residual regurgitation higher than 1 degree (scale 1 to 4) were excluded from the study.

Preoperative clinical and echocardiographic variables are showed in Table 1.

This study was approved by our institution's ethical committee/institutional review board.

### 2.2. Surgical Procedures

**Figure 1:**
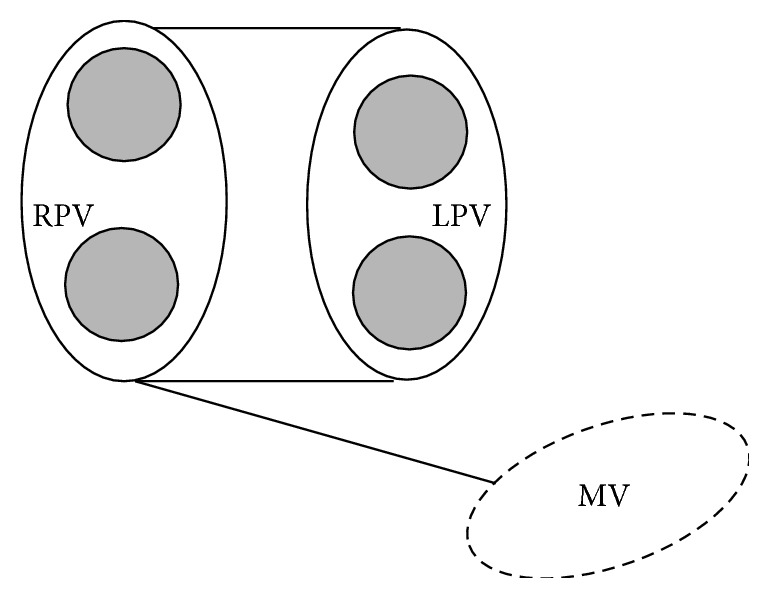
Operation schema of left Maze. After epicardial isolation of both pulmonary veins, we performed a double union line between the left and right pulmonary islands and a lesion connecting the right pulmonary one to the posterior annulus of the mitral valve at P2 or P3 portion (according to circumflex artery anatomy). LPV = left pulmonary veins. MV = mitral valve. RPV = right pulmonary veins.

RF energy was used to create continuous endocardial and epicardial lesions mimicking most of the left atrial incisions set as described in the Cox-Maze III procedure [12]. We did not perform any ablation line in the right atrium (Figure 1). In all patients a bipolar device was used (Cardioblate BP2 Irrigated RF Surgical Ablation System, Medtronic Inc., Minneapolis, MN, USA). The tip of the probe was irrigated with saline at room temperature at a flow rate of 4–6 mL/min.

Cardiopulmonary bypass was used with standard aortic cannulation, bicaval cannulation, and moderate hypothermia. After epicardial isolation of both pulmonary veins and of the left atrial appendage performed before aortic cross-clamping, some of the additional LA incisions sets currently used in the “cut and sew” technique were replaced by RF ablation lines, in particular represented by a double union line between the left and right pulmonary islands and a lesion connecting the right pulmonary one to the posterior annulus of the mitral valve at P2 or P3 portion (according to circumflex artery anatomy). In all patients the LA appendage was internally or externally closed.

After completion of the RF ablation, mitral valve repair or replacement was performed (Table 1).

### 2.3. Postoperative Management

Postoperative general care was similar to routine cardiac surgical procedures. Modality of amiodarone prophylaxis protocol treatment was the same in all patients, represented by iv amiodarone 1200 mg/24 h in the first postoperative day, followed by 200 mg per os every 8 hours until hospital discharge and then 200 mg daily for six months. In case of amiodarone intolerance or contraindications, propafenone was employed.

Patients showing SR or an ectopic atrial rhythm with rate inferior to 70 bpm in the first 48–72 postoperative hours were treated with temporary atrial pacing fixed at 80 bpm with the aim of avoiding supraventricular ectopic beats occurrence and consequently AF onset and of favoring atrial electrical activity recovery. Postoperative atrial tachyarrhythmias recurrences not responsive to medical treatment were submitted to direct-current (DC) shock; in this case, a loading dose of amiodarone was administrated before and until 24 hours after DC shock and then interrupted.

### 2.4. Clinical Follow-Up

Early postoperative rhythms were checked daily by standard 12-channel surface electrocardiogram. Follow-up 24-hour Holter monitoring was checked postoperatively at 3, 6, 12, and 24 months after the intervention.

### 2.5. Echocardiographic Follow-Up

Contemporary to clinical follow-up, all patients were evaluated with 2-dimensional transthoracic echocardiography by the same cardiologist at 3, 6, 12, and 24 months in order to specifically monitor the evolution of cardiac chambers dimensions and systolic performance and to record left and RA contractility presence. In more detail, biatrial transport function was checked in all patients, independently from the original (paroxysmal or persistent) AF type.

All echocardiographic examinations were performed with a Philips ultrasound system (iE33, Andover, MA, USA). LA diameter was measured at the time of aortic valve closure on the M-mode echocardiogram in the parasternal long-axis view. End-diastolic and end-systolic internal LV diameters were determined from the M-mode echocardiogram and fractional shortening was obtained.

Systolic PAP was calculated by adding transtricuspid pressure gradient to mean right atrial pressure estimated from inferior vena cava diameter and motion during respiration as follows: mean right atrial pressure was estimated to be 5 mmHg if there was complete collapse of a normal inferior vena cava during inspiration, 10 mmHg if a normal inferior vena cava collapse was >50%, 15 mmHg if a dilated inferior vena cava collapsed by >50% with inspiration, and 20 mmHg if there was no visible evidence of dilated inferior vena cava collapse.

Transmitral flow velocity was measured with pulsed Doppler echocardiography, with a sample volume positioned at the level of the mitral tip in the apical four-chamber view, and was recorded on a strip chart at a paper speed of 100 mm/s. Peak velocity and the time-velocity integral of the early filling wave (E wave) and of the late filling wave (A wave) were determined. A/E ratio, representing atrial contribution to ventricular diastolic filling, was obtained. Each measurement was obtained as an average of 6 to 8 consecutive beats. We considered that a peak A wave velocity ≥10 cm/s indicated echocardiographic evidence of effective atrial contraction [9].

### 2.6. Statistical Analysis

Data were collected and managed in Microsoft Excel 2003 and analyzed with SPSS 12.0 software (SPSS, Inc., Chicago, IL, USA). Continuous variables were presented as mean ± SD and categorical variables as percentages or numbers. Seventeen variables were evaluated univariately to identify predictors of arrhythmia recurrence and of AC restoration. Univariate analysis was performed for all relevant categorical variables by means of *λ* ^2^ analysis and Student's *t*-tests when indicated. A multivariate Cox proportional hazard model (including factors positive at univariate analysis) was used to determine the independent predictors of AC recuperation and of AF reappearance. A multiple events Cox model was applied too for taking into account the chance of multiple arrhythmia and transport loss recurrences.

Categoric and continuous variables associations were, respectively, investigated with *λ* ^2^ test and Spearman rank correlation coefficient.

McNemar test was employed for evaluating LA and RA contractility concordance.

A value of *p* less than 0.05 was considered statistically significant.

## 3. Results

### 3.1. Early Postoperative Data and 2-Year Rhythm Follow-Up

Mean aortic cross-clamp and cardiopulmonary bypass time were, respectively, 95 ± 38 and 121 ± 43 minutes. In all patients who underwent mitral valve repair, a residual valvular regurgitation lower than 1 degree (scale 1 to 4) was achieved.

One patient (0.8%) died in the Intensive Care Unit for unresponsive cardiac failure. Postoperative complications included major ischemic strokes (2/122, 1.6%) and reoperation for bleeding (4/122, 3.2%) whose origin was not related to Maze lesions.

79 patients (65%) were discharged from the hospital in SR, 29 (24%) presented with stable AF, and 5 showed an atrial flutter while 9 patients had an atrial ectopic rhythm with heart rate superior to 70 bpm.

2-year clinical follow-up was achieved in all patients. There were 2 (1.6%) late deaths (1 progressive congestive heart failure and 1 extra-cardiac cause). Stable SR was achieved in 96 patients (79%), while 18 (15%) experienced AF recurrence; most of them (15/18, 83%) were persistent arrhythmias resistant to pharmacological or electrical shock, and the others had AF episodes responsive to medical treatment or DC shock. No patients presented postincisional tachycardia. In the whole study population, 7 patients (5.7%) underwent permanent pacemaker implantation.

### 3.2. Contractility Restoration

**Figure 2:**
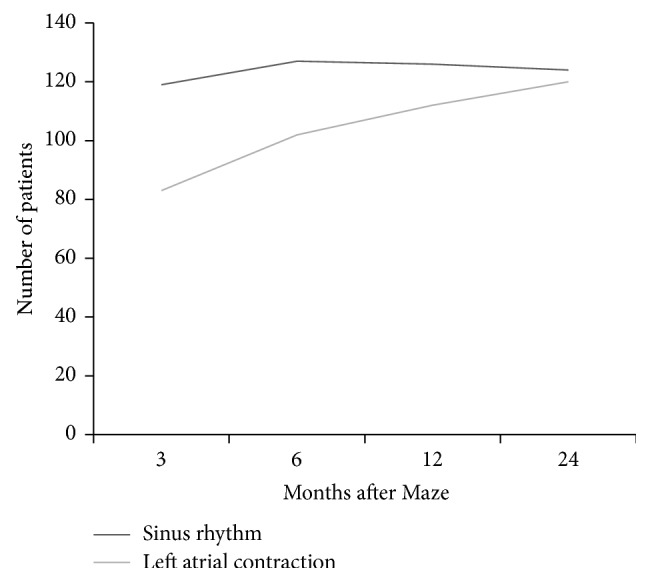
Sinus rhythm-left atrial contractility coexistence. The percentage of sinus rhythm-left atrial transport function coexistence increased progressively, joining about 98% two years after the operation.

At early follow-up (3 months), only 76% of patients showing a stable SR presented with a corresponding efficacious left AC. Nevertheless, such percentage of coexistence increased progressively, joining about 98% two years after the operation (Figure 2).

**Figure 3:**
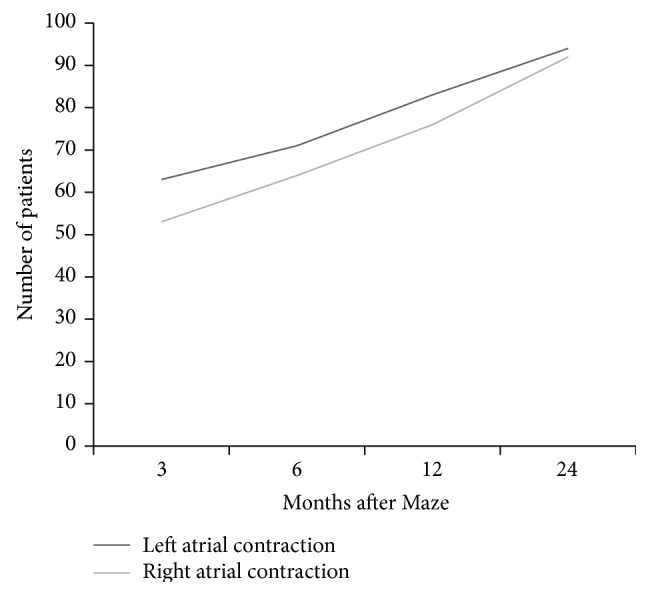
Biatrial contractility coexistence. At 3 months follow up, only in 84% of patients with an A wave both atria efficaciously contracted (Mc Nemar Kappa coefficient equal to 0.68), while 2 years after the intervention the percentage of bilateral “kick” coexistence reached 98% (Mc Nemar Kappa coefficient 0.87).

LA contraction failed to be accompanied in every case by a corresponding RA contraction, since, at 3 months of follow-up, only in 84% of patients with an “A” wave both atria efficaciously contracted (McNemar Kappa coefficient equal to 0.68), while 2 years after the intervention the percentage of bilateral “kick” coexistence reached 98% (McNemar Kappa coefficient 0.87) (Figure 3).

**Figure 4:**
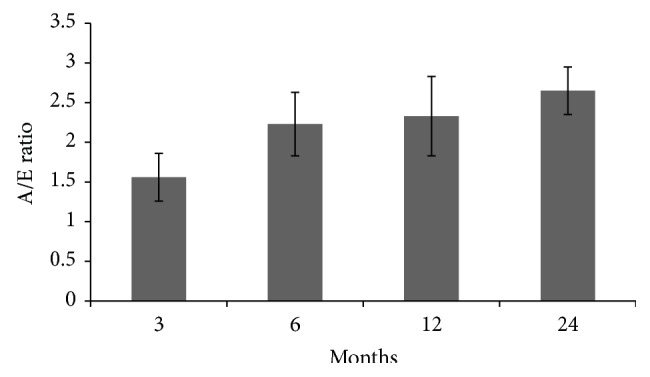
Left A/E ratio trend. Left A/E ratio slightly augmented until a mean value of 2.65 at 24 months of echocardiographic follow-up. Vertical bars denote 95% confidence intervals.

In patients who remained in persistent SR without known arrhythmia recurrence (nearly 74% of all population), left A/E ratio slightly augmented until a mean value of 2.65 at 24 months of echocardiographic follow-up (Figure 4).

### 3.3. Risk Factors Analysis of Maze Failure

The univariate analysis of the predictive factors of freedom from AF recurrence at 2-years of follow-up is shown in Table 2 and identifies as significant protective variables a preoperative arrhythmia duration inferior to 36 months (meaning the period of any AF type history: for longstanding one, it was calculated from the beginning date, but for paroxysmal arrhythmia from the date of the first episode recording), 3 and 6 months of left AC restoration, and SR presence at hospital discharge. Such last feature failed to be confirmed at multivariate analysis (Table 2).

Due to uniformity of antiarrhythmic prophylaxis and treatment in case of arrhythmia recurrence the item “amiodarone use” was not included in the statistical analysis of risk factors of Maze failure.

In order to better evaluate the theoretical possibility of several AF recurrences, a multiple events Cox model was applied, again revealing the protective role played by a shorter preoperative arrhythmia duration and by early AC regain.

### 3.4. Predictive Factors of LA Contractility Restoration

The same features used for uni- and multivariate analysis for freedom from AF recurrence (with the obvious replacement of 3 and 6 months of left AC by 3 and 6 months of SR presence for late AC regain evaluation) were entered into the statistical model in order to evaluate possible predictive factors for absence of left AC recovery. At multivariate analysis, significant predictors for absence of LA transport function restoring were preoperative arrhythmia duration for 6–12 and 24 months of follow-up (*p* value 0.005, 0.05, and 0.017, resp.) and basal PAP for 3 and 6 months of contractility (*p* value 0.02 and 0.03, resp.). 3- and 24-month results are shown in Tables 3 and 4. No other common clinical or operative features were highlighted in patients presenting SR without corresponding AC at different follow-up checks.

Again, a multiple events Cox model was applied, confirming the unfavorable role played by longer AF duration (*p* = 0.005) and by higher PAP (*p* = 0.029) (Table 5).

### 3.5. Contractility and Echocardiographic Measurements

Some statistically significant associations were retrieved between cardiac chambers dimensions and function and biatrial A/E ratios (considered as markers of contractility presence) evaluated at different follow-up times; in more detail (Table 6),

- minor LA dimensions were related with improved future A/E value and consequently with more pronounced LA contractility;
- 3-month augmented left A/E favored greater improvement of LA diameter evaluated 1 and 2 years after surgery;
- 3 and 24 months of better RA contractility corresponded to minor contemporary decrease of LVEF with respect to basal values;
- 12-month greater right A/E ratio was associated with decreased 24-month LV volumes and better LVEF.

## 4. Discussion

The association between AF and mitral valve disease is present in almost 60% of cases needing surgical correction. For more than 20 years, Cox-Maze procedure has been the gold standard for treating AF surgically [13]; despite its high success rate, it entails long surgery times and significant bleeding risks [14]. That is the reason why alternative energy sources and simplifications of ablation sets have been progressively developed, including irrigated RF. Such device achieves an acceptable (although inferior to the surgical Maze) degree of SR conversion at mid-long-term follow-up, equal to 45–95% [15–17].

If arrhythmia abolition has represented the most important goal of the procedure, it was even specifically conceived for allowing the entire atrial myocardium to be activated preserving postoperative atrial transport function [10]. The importance of AC is double, since, firstly, it may contribute to an increase in stroke volume, particularly in case of fast heart rates [18], and second, it is reasonable to think that the absence of an efficacious mechanical atrial action recovery even if associated with SR detection could favor intra-atrial thrombi formation and consequent thromboembolic systemic phenomena [19], thus making the decision to interrupt anticoagulant treatment unsafe.

Based upon such considerations, we decided to focus our attention onto biatrial transport function behavior in a relatively homogeneous sample of patients affected by mitral valve disease and concomitant AF in whom RF modified left Maze procedure obtained 2-year arrhythmia abolition in about 80% of cases, in agreement with what is reported in medical literature.

Previous studies retrieved that restoration of both SR and atrial contraction varies from 21% to 95%, depending on AF etiology [20–25]. In more detail, in case of exclusively LA Maze ablation, it has been proved that, despite successful SR restoration, the progressive loss of LA function as well as LV diastolic function is more prominent in patients affected by rheumatic disease with respect to those with degenerative prolapse [26].

In our trial, AC restoring was a dynamic and raising process, a finding that perhaps translates into the practical advice of persisting in serially controlling by echocardiography also patients that early experience SR reestablishment few months after the procedure, because in about three-quarters of cases LA is still “stunned.” This consideration may assume a great empirical role, since it discourages from interrupting anticoagulant and antiarrhythmic treatment too early, even in presence of detected stable SR and low CHADS2 score.

Another element that could support this prudent attitude is the evidence of progressive increase of atrial contribution entity. A/E ratio time-course after Maze is a discussed question, as some authors [27] describe a decreasing trend but others [10] a fluctuant tendency more in line with our findings. On the contrary, Yuda and colleagues [28] demonstrated that once AC was resumed, its degree did not change during follow-up: the difference with respect to our results may lie in the major prevalence in their sample of mitral valve replacement (prosthesis could modify modality of LA transport function) and of basal LV dysfunction associated with a more aggressive lesions set performed with mixed cut and sew technique and cryoenergy. A recent report from Sayed et al. [29] highlighted the fact that the “left side only” ablation procedure, when compared to biatrial one, allowed better 6-month A/E ratio but with a mean value largely lower (0.52) than the one emerging from our trial. Nevertheless, again, enrolled patients were all affected by rheumatic disease, thus perhaps explaining worse postoperative atrial function.

Starting from the assumption that SR and AC recovery represent two inseparable arms of the same question, we tried to deeply examine possible predictors of restoration and maintenance of atrial transport function at different intervals, with the objective of screening patients who could fully benefit from the procedure. Moreover, we applied a supplementary statistical tool addressed at also evaluating the intrinsic arrhythmia nature consisting in its chance of several recurrent episodes. In fact, it is well known that many subjects experience repeated heart rhythm changes (and consequently disappearance of atrial “kick”) above all in the first postoperative months, probably due to recovery process, to postprocedural inflammation status, or to altered neurohumoral mechanisms [30].

The only shared negative predictive factor for both SR and left AC restoring highlighted by our analysis was preoperative AF duration, with a cut-off identified at 36 months. If the role of longstanding arrhythmia largely emerged in medical literature [31, 32], other issues were unexpectedly retrieved: in more detail, early atrial transport function regain was found to strictly correlate with future stable freedom from arrhythmia recurrence, while basal PAP influenced the possibility of left AC recuperation in the early postoperative phases and thus the symmetrical Maze goal of SR achievement. Concerning the former association, to the best of our knowledge, this represents the first report in medical literature, but, interestingly, the reverse interrelation failed to be retrieved. We may speculate that AC is a secondary phenomenon which follows SR recovery (and not in all cases) and which is consequently something of more definitive favoring perpetuation of SR, while early SR appearance could be a temporary event not necessarily implying rhythm and atrial transport function stability.

The discouraging role played by basal PAP onto future atrial transport function recuperation was previously reported only by Reyes et al. [33], but without statistical significance. Its real implication needs surely further analysis but may perhaps reflect the fact that fixed and longstanding mitral pathology associated with chronic AF which have already caused progressive augmentation of pulmonary pressures with effects onto pulmonary circulation represent a more serious and advanced disease stage implying more possibilities of Maze failure. A deeper investigation of which type of pulmonary hypertension is involved (pre- or postcapillary, in order to understand if its adverse role is more due to a pulmonary or cardiac component) is required but perhaps arises the doubt whether a more comprehensive patient's evaluation including right heart performance should be performed.

Medical literature extensively addressed the problem of identifying predictors able to discourage the surgeon to perform ablation, including old age, larger LA diameter, lower amplitude of p-wave, having a rheumatic mitral valve disease, permanent AF, and lesion sets of Maze procedures [32, 34]. In case of analysis specifically focused onto restoration of atrial contraction, AF duration, LA diameter, an history of hypertension, rheumatic disease, and the presence of prosthetic valve failure arose as significant negative predictors [25, 26, 35]. In our opinion, all these features reflect the negative influence played by arrhythmia “chronic state” onto Maze success; the same conclusion can be suggested by our data too, since PAP and longer preoperative arrhythmia duration are intimately linked with the idea of a longstanding phenomenon. Based upon such concept, we can perhaps draw the suggestion of carefully screening patients potentially candidates to surgical ablation, being aware that those presenting “ancient” arrhythmia with augmented PAP will unlikely benefit from the procedure.

Nevertheless, in our analysis, LA dimensions did not represent a negative predictor of SR-AC restoring; however, such absence of relationship is partially mitigated by the finding of a bidirectional association between LA size and chance of future AC recovery or, at the opposite, between early atrial “kick” recuperation and more marked future atrial diameter and area decrease. To the best of our knowledge, this is the first demonstration of a strength link between these two elements which are logically and theoretically related but whose modality of respective behavior was not completely clear.

Moreover, we found that the atria seem to differently regain contraction after Maze. Available data addressing the question show various rates of RA function recovery [10, 33]. Although it is not directly touched, we can imagine that RA is somehow influenced by the ablation procedure itself. Such subject may represent an interesting matter also considering that, in our study, RA transport function recuperation appears to favor better contemporary and future LV contractile performance and decreased volumes. Even if these findings have not been found at all follow-up intervals, they may represent an intriguing hypothesis of a supplementary interrelation between right and left cardiac sides leading to the suggestion of reconsidering RA role.

An important study limitation is represented by the limited sample size which does not allow drawing definitive conclusions; we believe that our preliminary results should be confirmed in larger trials including “more ancient” mitral and fibrillating patients, in order to better describe the negative influence of longstanding arrhythmia onto Maze objectives.

## 5. Conclusions

We serially assessed biatrial function after RF Maze procedure in patients affected by mitral valve disease.

In the early postoperative phases, a regular rhythm with P-waves was accompanied by a coexisting atrial transport function only in three quarters of cases, but concordance joined about 100% at two years of follow-up. Once LA contraction was resumed, its degree augmented progressively and was associated with a corresponding RA transport function in an increasing percentage of cases at late follow-up.

A strict interaction was detected between LA chamber postoperative evolution and LA contractility recovery, as a decrease of LA size favored atrial “kick” reappearance and vice versa.

SR and AC appear to be two intimately linked objectives of the Maze procedure, since early atrial transport function recuperation favors future stable freedom from arrhythmia recurrence.

Our study highlighted the deleterious influence of arrhythmia “chronic state” onto procedural success and the importance of the cardiac right side, an entity somehow overlooked: even if these data need further investigation in order to elucidate a possible clinical implication too, we retrieved that, first, preoperative arrhythmia duration and, more interesting, pulmonary hypertension (an element that could be associated with a latent right ventricular dysfunction) play a role in predicting Maze failure in terms of SR-AC restoring and, second, RA contractility has an influence on LV contractile performance recovery and volumes decrease.

## Figures and Tables

**Table 1:** Patients characteristics.

	*n* = 122
Sex (M : F)	59/63
Age (years)	62 ± 8.5
Chronic AF	79 (64.7%)
Paroxysmal AF	53 (35.3%)
AF duration (months)	28.3 (range 1–220)
LAD (mm)	56 ± 12
LAA (cm^2^)	34 ± 7
LVEDD (mm)	56 ± 8
LVESD (mm)	34 ± 9
PAP (mmHg)	33 ± 15
LVEF (%)	57 ± 9
Mitral stenosis	35 (39%)
Mitral regurgitation	87 (71%)
Mitral valve repair	76 (62%)
Mitral valve replacement	46 (38%)

AF, atrial fibrillation; LAD, left atrial diameter; LAA, left atrial area; LVEDD, left ventricular end-diastolic diameter; LVESD, left ventricular end-systolic diameter; LVEF, left ventricular ejection fraction; M : F, males : females.

**Table 2:** Univariate and multivariate analysis for freedom from AF recurrence at 2-year follow-up.

Factors	Univariate *p* value	Multivariate analysis		
		HR	95% CI	*p* value
AF duration (>36 months)	<0.0001	1.402	1.13–1.78	0.02
3-month left AC restoration	<0.0001	1.238	1.07–1.52	0.03
6-month left AC restoration	<0.0001	1.127	1.05–1.48	0.04
AF at discharge	0.003	0.841	0.49–1.23	0.21
Cross-clamp time	0.13			
Age	0.23			
Rheumatic disease	0.13			
PAP	0.09			
Sex	0.25			
Permanent/persistent AF	0.18			
Postoperative bleeding	0.26			
CPB time	0.24			
LAD (mm)	0.17			
LAA (cm^2^)	0.12			
LVESD (mm)	0.28			
LVEDD (mm)	0.25			
LVEF (%)	0.31			

Not all factors were entered into multivariate model.

AF, atrial fibrillation; CI, confidence interval; CPB, cardiopulmonary bypass; HR, hazard ratio; LAA, left atrial area; LAD, left atrial diameter; LVEDD, left ventricular end-diastolic diameter; LVEF, left ventricular ejection fraction; LVESD, left ventricular end-systolic diameter; PAP, pulmonary artery pressure.

**Table 3:** Univariate and multivariate analysis for absence of LA contractility recovery at 3 months of follow-up.

Factors	Univariate *p* value	Multivariate analysis		
		HR	95% CI	*p* value
PAP	<0.0001	1.05	1.01–1.11	0.02
LAA (cm^2^)	0.002	1.06	1.01–1.14	0.06
Duration of AF	0.003	1.014	1–1.03	0.06
Age	0.23			
Rheumatic disease	0.12			
Sex	0.14			
Permanent/persistent AF	0.18			
Postoperative bleeding	0.47			
CPB time	0.31			
Cross-clamp time	0.19			
AF at discharge	0.17			
LAD (mm)	0.08			
LVESD (mm)	0.28			
LVEDD (mm)	0.73			
LVEF (%)	0.58			

Not all factors were entered into multivariate model.

AF, atrial fibrillation; CI, confidence interval; CPB, cardiopulmonary bypass; HR, hazard ratio; LAA, left atrial area; LAD, left atrial diameter; LVEDD, left ventricular end-diastolic diameter; LVEF, left ventricular ejection fraction; LVESD, left ventricular end-systolic diameter; PAP, pulmonary artery pressure.

**Table 4:** Univariate and multivariate analysis for absence of LA contractility recovery at 24 months of follow-up.

Factors	Univariate *p* value	Multivariate analysis		
		HR	95% CI	*p* value
Duration of AF	<0.0002	1.034	1.006–1.063	0.017
3-month SR presence	0.07			
6-month SR presence	0.08			
LAA (cm^2^)	0.09			
PAP	0.08			
Age	0.26			
Rheumatic disease	0.31			
Sex	0.18			
Permanent/persistent AF	0.12			
Postoperative bleeding	0.59			
CPB time	0.64			
Cross-clamp time	0.33			
AF at discharge	0.24			
LAD (mm)	0.12			
LVESD (mm)	0.35			
LVEDD (mm)	0.78			
LVEF (%)	0.49			

Not all factors were entered into multivariate model.

AF, atrial fibrillation; CI, confidence interval; CPB, cardiopulmonary bypass; HR, hazard ratio; LAA, left atrial area; LAD, left atrial diameter; LVEDD, left ventricular end-diastolic diameter; LVEF, left ventricular ejection fraction; LVESD, left ventricular end-systolic diameter; PAP, pulmonary artery pressure.

**Table 5:** Multiple events Cox model for absence of LA contractility recovery.

Factors	HR	95% CI	*p* value
Duration of AF (>36 months)	1.14	1.07–1.22	0.005
PAP	1.12	1.04–1.21	0.029

AC, atrial contractility; AF, atrial fibrillation; CI, confidence interval; HR, hazard ratio; PAP, pulmonary artery pressure.

**Table 6:** Contractility and echocardiographic measurements (Spearman rank correlation).

Factors	*p* value
3 months of LAD–6 months of left A/E	0.035
3 months of LAA–6 months of left A/E	0.023
3 months of LAD–12 months of left A/E	0.033
6 months of LAA–12 months of left A/E	0.033
6 months of LAA–24 months of left A/E	0.023
3 months of left A/E–12 months of LAD	0.009
3 months of left A/E–24 months of LAD	0.029
3 months of right A/E–3 months of delta LVEF	0.035
24 months of right A/E–24 months of delta LVEF	0.044
12 months of right A/E–24 months of LVEDD	0.033
12 months of right A/E–24 months of LVEF	0.022

LAD, left atrial diameter; LAA, left atrial area; LVEDD, left ventricular end-diastolic diameter; LVEF, left ventricular ejection fraction.

Delta LVEF means the difference between follow-up time LVEF and basal LVEF.

## References

[1] Deneke T., Khargi K., Grewe P. H. (2002). Efficacy of an additional MAZE procedure using cooled-tip radiofrequency ablation in patients with chronic atrial fibrillation and mitral valve disease: a randomized, prospective trial. *European Heart Journal*.

[2] Cox J. L., Schuessler R. B., D'Agostino H. J. (1991). The surgical treatment of atrial fibrillation. III. Development of a definitive surgical procedure. *Journal of Thoracic and Cardiovascular Surgery*.

[3] Bakir I., Casselman F. P., Brugada P. (2007). Current strategies in the surgical treatment of atrial fibrillation: review of the literature and Onze Lieve Vrouw Clinic’s strategy. *Annals of Thoracic Surgery*.

[4] von Oppell U. O., Masani N., O'Callaghan P., Wheeler R., Dimitrakakis G., Schiffelers S. (2009). Mitral valve surgery plus concomitant atrial fibrillation ablation is superior to mitral valve surgery alone with an intensive rhythm control strategy. *European Journal of Cardio-Thoracic Surgery*.

[5] McCarthy P. M., Cosgrove D. M., Castle L. W., White R. D., Klein A. L. (1993). Combined treatment of mitral regurgitation and atrial fibrillation with valvuloplasty and the maze procedure. *The American Journal of Cardiology*.

[6] Sie H. T., Beukema W. P., Elvan A., Misier A. R. R. (2004). Long-term results of irrigated radiofrequency modified maze procedure in 200 patients with concomitant cardiac surgery: six years experience. *The Annals of Thoracic Surgery*.

[7] Kawaguchi A. T., Kosakai Y., Sasako Y., Eishi K., Nakano K., Kawashima Y. (1996). Risks and benefits of combined maze procedure for atrial fibrillation associated with organic heart disease. *Journal of the American College of Cardiology*.

[8] Tinetti M., Costello R., Cardenas C., Piazza A., Iglesias R., Baranchuk A. (2012). Persistent atrial fibrillation is associated with inability to recover atrial contractility after MAZE IV surgery in rheumatic disease. *Pacing and Clinical Electrophysiology*.

[9] Manning W. J., Leeman D. E., Gotch P. J., Come P. C. (1989). Pulsed Doppler evaluation of atrial mechanical function after electrical cardioversion of atrial fibrillation. *Journal of the American College of Cardiology*.

[10] Albirini A., Scalia G. M., Daniel Murray R. (1997). Left and right atrial transport function after the maze procedure for atrial fibrillation: an echocardiographic doppler follow-up study. *Journal of the American Society of Echocardiography*.

[11] Fuster V., Rydén L. E., Cannom D. S. (2011). 2011 ACCF/AHA/HRS focused updates incorporated into the ACC/AHA/ESC 2006 guidelines for the management of patients with atrial fibrillation: a report of the American College of Cardiology Foundation/American Heart Association Task Force on Practice Guidelines. *Circulation*.

[12] Cox J. L., Ad N., Palazzo T. (2000). Current status of the Maze procedure for the treatment of atrial fibrillation. *Seminars in Thoracic and Cardiovascular Surgery*.

[13] Prasad S. M., Maniar H. S., Camillo C. J. (2003). The Cox maze III procedure for atrial fibrillation: long-term efficacy in patients undergoing lone versus concomitant procedures. *Journal of Thoracic and Cardiovascular Surgery*.

[14] Bauer E. P., Szalay Z. A., Brandt R. R. (2001). Predictors for atrial transport function after mini-maze operation. *Annals of Thoracic Surgery*.

[15] Fujita T., Kobayashi J., Toda K. (2010). Long-term outcome of combined valve repair and maze procedure for nonrheumatic mitral regurgitation. *Journal of Thoracic and Cardiovascular Surgery*.

[16] Lazopoulos G., Mihas C., Manns-Kantartzis M. (2014). Radiofrequency ablation for atrial fibrillation during concomitant cardiac surgery: mid-term results. *Herz*.

[17] Kobayashi J., Sasako Y., Bando K. (2002). Eight-year experience of combined valve repair for mitral regurgitation and maze procedure. *Journal of Heart Valve Disease*.

[18] Benchimol A. (1969). Significance of the contribution of atrial systole to cardiac function in man. *The American Journal of Cardiology*.

[19] Cox J. L., Ad N., Palazzo T. (1999). Impact of the maze procedure on the stroke rate in patients with atrial fibrillation. *Journal of Thoracic and Cardiovascular Surgery*.

[20] Feinberg M. S., Waggoner A. D., Kater K. M., Cox J. L., Lindsay B. D., Pérez J. E. (1994). Restoration of atrial function after the maze procedure for patients with atrial fibrillation: assessment by Doppler echocardiography. *Circulation*.

[21] Sandoval N., Velasco V. M., Orjuela H. (1996). Concomitant mitral valve or atrial septal defect surgery and the modified Cox-maze procedure. *The American Journal of Cardiology*.

[22] Cox J. L., Boineau J. P., Schuessler R. B., Kater K. M., Lappas D. G. (1993). Five-year experience with the maze procedure for atrial fibrillation. *Annals of Thoracic Surgery*.

[23] Kobayashi J., Yamamoto F., Nakano K., Sasako Y., Kitamura S., Kosakai Y. (1998). Maze procedure for atrial fibrillation associated with atrial septal defect. *Circulation*.

[24] Izumoto H., Kawazoe K., Kitahara H. (1997). Can the maze procedure be combined safely with mitral valve repair?. *Journal of Heart Valve Disease*.

[25] Yuda S., Nakatani S., Isobe F., Kosakai Y., Miyatake K. (1998). Comparative efficacy of the maze procedure for restoration of atrial contraction in patients with and without giant left atrium associated with mitral valve disease. *Journal of the American College of Cardiology*.

[26] Kim H. W., Moon M. H., Jo K. H., Song H., Lee J. W. (2015). Left atrial and left ventricular diastolic function after the maze procedure for atrial fibrillation in mitral valve disease: degenerative versus rheumatic. *Indian Journal of Surgery*.

[27] Lönnerholm S., Blomström P., Nilsson L., Blomström-Lundqvist C. (2008). Long-term effects of the maze procedure on atrial size and mechanical function. *Annals of Thoracic Surgery*.

[28] Yuda S., Nakatani S., Kosakai Y., Yamagishi M., Miyatake K. (2001). Long-term follow-up of atrial contraction after the maze procedure in patients with mitral valve disease. *Journal of the American College of Cardiology*.

[29] Sayed S. A., Katewa A., Srivastava V., Jana S., Patwardhan A. M. (2014). Modified radial v/s biatrial maze for atrial fibrillation in rheumatic valvular heart surgery. *Indian Heart Journal*.

[30] de Lima G. G., Kalil R. A. K., Leiria T. L. L. (2004). Randomized study of surgery for patients with permanent atrial fibrillation as a result of mitral valve disease. *Annals of Thoracic Surgery*.

[31] Baek M.-J., Na C.-Y., Oh S.-S. (2006). Surgical treatment of chronic atrial fibrillation combined with rheumatic mitral valve disease: effects of the cryo-maze procedure and predictors for late recurrence. *European Journal of Cardio-Thoracic Surgery*.

[32] Gaynor S. L., Schuessler R. B., Bailey M. S. (2005). Surgical treatment of atrial fibrillation: predictors of late recurrence. *Journal of Thoracic and Cardiovascular Surgery*.

[33] Reyes G., Benedicto A., Bustamante J. (2009). Restoration of atrial contractility after surgical cryoablation: clinical, electrical and mechanical results. *Interactive Cardiovascular and Thoracic Surgery*.

[34] Itoh A., Kobayashi J., Bando K. (2006). The impact of mitral valve surgery combined with maze procedure. *European Journal of Cardio-Thoracic Surgery*.

[35] Kim Y.-J., Sohn D.-W., Park D.-G. (1998). Restoration of atrial mechanical function after maze operation in patients with structural heart disease. *American Heart Journal*.

